# The Effect of the Partial Baking Method on the Quality, Volatile Compounds Profile, and Glycemic Index of Some Traditional Turkish Bread Types

**DOI:** 10.3390/foods15132392

**Published:** 2026-07-05

**Authors:** Sena Yilmaz, Hatice Bekiroglu, Gorkem Ozulku

**Affiliations:** 1 Department of Food Engineering, Faculty of Chemical and Metallurgical Engineering, Yildiz Technical University, Istanbul 34220, Türkiye; 2 Department of Food Engineering, Faculty of Agriculture, Sirnak University, Sirnak 73300, Türkiye

**Keywords:** frozen storage, textural characteristics, staling rate

## Abstract

This study evaluated the effects of the partial baking (par-baking) method on bread quality, staling properties, volatile compound profile, and estimated glycemic index (eGI) of some traditional Turkish bread types, and compared the results with those of regular bread (RB). Bread samples resembling Afyonkarahisar potato bread and Vakfıkebir bread were produced under laboratory conditions and named Afyonkarahisar potato style bread (APSB) and Vakfıkebir style bread (VKSB). APSB and VKSB were subjected to par-baking (7 min at 220 °C for APSB and 9 min at 220 °C for VKSB), followed by frozen storage at −18 °C for up to 60 days and final baking. Also, quality changes in the breads after final baking were assessed over 5 days (days 0, 1, 3, and 5) and compared with conventionally baked control samples. The reduction in specific volume due to par-baking was more pronounced in regular bread than in the traditional bread samples. Hardness values of par-baked breads increased significantly after 60 days of frozen storage for all bread types (*p* < 0.05). Storage of breads after final baking caused substantial changes in textural properties, but no significant differences were observed in terms of staling rate among the non-par-baked samples during 5 days of storage at room temperature (*p* = 0.078). VKSB consistently exhibited the lowest eGI values for both the non-par-baked group and the par-baked group. Principal component analysis (PCA) clearly separated the volatile compound profiles of non-par-baked breads (VKSB-Day 0 and APSB-Day 0) from those of regular bread and par-baked breads. The findings of this study suggest that par-baking technology is a promising approach for breads similar to traditional breads, enabling wider consumption without marked quality deterioration up to 30 days and resulting in a lower estimated glycemic index (eGI) than regular bread.

## 1. Introduction

Bread is one of the most widely consumed staple foods worldwide, with an average annual per capita consumption of 59–70 kg. In Türkiye, bread consumption is among the highest in the world, averaging 104 kg per capita per year [1,2]. However, bread undergoes rapid quality deterioration during storage. This deterioration, commonly referred to as staling, directly affects both sensory properties and consumer acceptability [3]. Staling is a complex phenomenon that cannot be explained by a single mechanism. Starch retrogradation, particularly the recrystallization of amylopectin chains, plays a central role in this process, while water redistribution within the bread matrix and changes in the gluten network further accelerate quality loss, such as crumb hardening, reduced elasticity, and increased crumbliness [4]. In this context, par-baking and frozen storage technologies have emerged as effective strategies for maintaining bread quality.

Partial baking (par-baking) is a bread-making method similar to conventional baking. However, during the baking stage, the product is only partially baked rather than fully baked. In this process, the dough is baked until the crumb structure is set, without significant crust formation or browning. After storage, it undergoes final baking to produce a fully baked product with a crispy crust. Final baking can be carried out in bakeries, supermarkets, hotels, restaurants, or even at home just before consumption. This technology enables the delivery of fresh bread to consumers while providing flexibility in production and distribution [5,6].

The changes occurring during bread storage affect not only the physical quality of bread but also its nutritional properties. The formation of resistant starch during starch retrogradation reduces starch digestibility and consequently lowers the glycemic index (GI) of bread [7]. Therefore, controlling starch retrogradation through processing and storage conditions has been recognized as an effective physical strategy for developing low-glycemic-index (low-GI) wheat bread. This effect is attributed to the formation of a more compact and ordered starch structure, which limits enzymatic accessibility and reduces starch digestibility [8]. However, starch retrogradation is also associated with undesirable quality changes, including crumb firming and loss of freshness during storage. Therefore, maintaining desirable technological properties while promoting resistant starch formation remains a significant challenge. Sourdough fermentation has been reported as a promising approach to mitigate these quality losses [9], and it is traditionally used in the production of many bread varieties.

Traditional bread types differ from regular bread types in terms of formulation and processing, which also influence their volatile compound profile, storage behavior, and nutritional characteristics. Especially, the volatile compound profile is their distinguished property. From this perspective, Mantou (Chinese steamed bread) and Altamura bread (traditional Italian bread) are widely studied examples [10,11,12]. A common characteristic of these breads is the use of sourdough, similar to Vakfikebir bread (originating from the Trabzon Province of Türkiye) and Afyonkarahisar potato bread (Afyonkarahisar, Türkiye). These breads were registered by the Turkish Patent and Trademark Office and received geographical indication status in 2018 [13,14]. Kotancilar et al. [15] investigated the staling properties of Vakfıkebir bread and reported that its prolonged freshness was associated with the high residual sugar and moisture content of the crumb, resulting from the low baking temperature and extended baking time. Afyonkarahisar potato bread showed no visible spoilage during 10 days of storage in a study of Yalçın [16]. Despite these advantages, studies integrating traditional formulations with modern processing technologies remain limited. Although par-baking and frozen storage have been studied individually, studies addressing their combined effects on both staling behavior and glycemic index in traditional breads are still relatively scarce. Therefore, this study aimed to investigate the effects of the par-baking method on the quality characteristics, staling behavior, volatile compound profile, and glycemic index of some bread types similar to traditional Turkish breads to facilitate their industrial-scale production and increase their consumption.

## 2. Materials and Methods

### 2.1. Materials

Wheat flour (12.99% moisture, 10.93% protein, 0.62% ash, and 57.1% water absorption) was purchased from Bonatellı Food Industry and Trade Inc. Co. (Balıkesir, Türkiye). Dry yeast (Pakmaya, İzmit, Türkiye), salt (Billur, İzmir, Türkiye), and potato (*Solanum tuberosum*) were purchased from a local market in Istanbul. Amyloglucosidase (from *Aspergillus niger*, 3330 U/mL) and glucose oxidase-peroxidase (GOPOD) reagent were purchased from Megazyme (Megazyme Int. Ltd., Wicklow, Ireland). Pepsin (from porcine gastric mucosa, P7000, activity > 250 U/mg), pancreatin (from porcine pancreas, P7545, 8 × USP specifications), and all other chemicals and solvents were purchased from Sigma-Aldrich (St. Louis, MO, USA).

### 2.2. Sourdough Production

Type I sourdough was prepared by mixing 30 g of refined flour and 30 g of tap water in a dough mixer (KitchenAid, Benton Harbor, MI, USA) for 5 min to obtain a dough yield (DY, [(amount of flour + water)/(amount of flour) × 100]) of 200. The dough was fermented in a fermentation cabinet (Nuve TK 252, Ankara, Türkiye) at 26 °C for 24 h. Daily propagation was carried out by adding flour and water in amounts equivalent to the dough produced on the previous day, while maintaining a constant DY of 200. The sourdough was propagated for three consecutive days under the same fermentation conditions (26 °C for 24 h). At the end of the propagation period, the mature type I sourdough was stored under refrigeration (4–7 °C), and periodic refreshment was carried out at one-week intervals.

### 2.3. Sourdough Analysis

The pH of the mature sourdough and weekly refreshed sourdough was measured using a pH meter (WTW Inolab 7110, Istanbul, Türkiye). Microbiological analyses were performed to determine lactic acid bacteria (LAB) and yeast populations. For this purpose, sourdough samples were homogenized in a stomacher with 0.1% (*w*/*v*) sterile buffered peptone water (Merck, Darmstadt, Germany) at a ratio of 1:9 (sourdough:BPW). The resulting serial dilutions were plated on Man, Rogosa, and Sharpe (MRS) agar for LAB enumeration and Malt Extract Agar for yeast enumeration. The plates were incubated at 37 °C for 48 h for LAB and at 30 °C for 72 h for yeast. Microbial counts were expressed as log CFU/g.

### 2.4. Production of Breads

Regular bread samples were prepared according to the American Association of Cereal Chemists 10-10B method [17], with some modifications. The formulation included 100 g of wheat flour, 1.5 g of salt, 2 g of dry yeast, and an appropriate amount of water determined according to the Farinograph water absorption value [17]. All ingredients were mixed in a dough mixer (KitchenAid, Benton Harbor, MI, USA) at speed 5 for 10 min. The dough pieces (165 g each) were shaped and placed in a proofing cabinet (85% RH, 30 °C). The dough samples were degassed after the initial 30 min of fermentation and subjected to an additional 30 min of fermentation. Following the second fermentation period, the final proofing was carried out for 55 min. Baking was performed in an electrical oven (Maksan, Nevşehir, Türkiye) at 210–220 °C for 21 min for non-par-baked samples. The formulation of the par-baked bread was identical to that of the non-par-baked bread; however, it was subjected to a partial baking process of 7 min. Par-baking conditions were determined through preliminary experiments. Following partial baking, the breads were stored at −18 °C for 15, 30, and 60 days. After storage, the samples were thawed at 26 °C for 1 h and subsequently subjected to a final baking step for 14 min to complete the baking process. All bread analyses were conducted after they were allowed to equilibrate at room temperature for 2 h.

The production process of Afyonkarahisar potato style bread (APSB) was revised with reference to the technical descriptions provided in Geleneksel Ekmeklerimiz by Köksel H. and Şanlıer N., published by Ankara Metropolitan Municipality Halk Ekmek [18]. Potatoes were boiled, mashed, and held for 24 h before use under refrigerated conditions (4 °C). The potato puree (40%, *w*/*w*) was blended with sourdough (20%, *w*/*w*), water (50%, *w*/*w*), salt (1.5%, *w*/*w*), and flour to obtain a homogeneous dough. The dough mass was fermented for 60 min in a proofing cabinet (80% RH, 30 °C). It was then divided into 165 g portions, molded, and placed on baking trays. Subsequently, the dough pieces were subjected to a final fermentation for an additional 60 min under the same conditions. All fermented doughs were baked at 210–220 °C for 21 min. The partial and final baking times and temperatures of the par-baked APSB were identical to those used for the par-baked RB.

Vakfıkebir style bread (VKSB) samples were prepared according to the method reported by Kotancilar et al. [13], with modifications for laboratory conditions. After the activation of the first and second sourdough phases, the main dough components (flour, water, salt, and yeast) were incorporated. The dough was kneaded for 10 min and subjected to bulk fermentation for 2 h. Following the rounding process, a final proofing period of 100 min was applied. The proofed samples were divided into two processing groups. The formulation of the par-baked breads followed that of the Vakfıkebir bread, employing a 9 min initial baking time at 210–220 °C. The par-baked products were subsequently stored at –18 °C for 15, 30, or 60 days. Prior to the final baking step, the samples were thawed at 26 °C for 60 min. Final baking was conducted for 14 min at 210–220 °C. All bread analyses were carried out after a 2 h equilibration period at room temperature.

**Figure 1:**
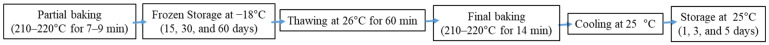
Flowchart of the par-baking process.

Two independent dough batches were produced for each bread type. The par-baking process was illustrated in Figure 1.

### 2.5. Moisture Content

The moisture content of the breads was determined using an infrared moisture analyzer (Radwag Balances and Scales, Radom, Poland). Crumb samples of approximately 0.5 g were placed on an aluminum pan and heated to 105 °C. The percentage mass loss corresponding to moisture evaporation was recorded. Measurements were performed under ambient conditions (23.9 ± 0.1 °C and 34.6 ± 0.3% RH), and the mean and standard deviation were calculated by performing two technical replicates for each sample.

### 2.6. Specific Volume and Baking Loss

The loaf volume (mL) was determined by using the rapeseed displacement technique [19]. Triplicate technical measurements were performed for each bread type. The specific volume was then obtained by expressing bread volume (mL) relative to its weight (g).

Baking loss is typically calculated as the percentage of weight lost by the bread during the baking process and calculated using Equation (1).

(1) $$Bakingloss\left(\%\right)=\frac{W_{1}-W_{2}}{W_{1}}\times 100$$

In this equation, $W_{1}$ represents the weight of the dough before baking, while $W_{2}$ represents the weight of the bread after baking and cooling. The weight of each dough was measured in triplicate.

### 2.7. Texture Profile Analysis

Following a 2 h equilibration period at room temperature, each bread sample was sliced into sections of 1.25 mm thickness. Texture Profile Analysis (TPA) was carried out using a texture analyzer (SMS TA.XT2 Plus, Glasgow, UK) equipped with a 5 kg load cell and a cylindrical compression probe of 36 mm diameter. The TPA measurements were performed at 50% compression with a test speed of 5.0 mm/s and a 5 s interval between the two compression cycles. All measurements were conducted in duplicate for each sample as technical replicates.

The bread samples after final baking were stored in polyethylene bags at room temperature for 5 days. The staling rate was calculated based on the ratio of the increase in crumb hardness to the storage duration (5 days), as described by Li et al. [20] using Equation (2).

(2) $$Stalingrate=\frac{H_{t}-H_{0}}{t}$$

$H_{t}$: Hardness value on the day.

$H_{0}$: Initial hardness value at day 0 (fresh bread).

t: Total storage duration (days).

### 2.8. Differential Scanning Calorimetry (DSC)

The enthalpy associated with water evaporation of bread samples was analyzed using a differential scanning calorimeter (DSC Q20, TA Instruments, New Castle, DE, USA) following the method reported by Kerch et al. [21]. Approximately 10 mg of each bread sample was accurately weighed and placed into hermetically sealed aluminum DSC pans to prevent moisture loss prior to analysis. An empty, hermetically sealed aluminum pan was used as the reference. The samples were heated from 25 °C to 200 °C at a constant heating rate of 10 °C min^−1^ under a nitrogen atmosphere. Heat flow was recorded as a function of temperature, and the enthalpy of water evaporation was calculated from the area under the endothermic peak. All measurements were performed in duplicate for each sample as technical replicates.

### 2.9. Glycemic Index

The digestion of the samples was carried out following the procedure described by Englyst et al. [22]. Briefly, 100 mg of each sample was transferred into 50 mL tubes containing 10 glass beads (5 mm in diameter). Subsequently, 2 mL of hydrochloric acid (0.05 M) supplemented with pepsin (5 mg/mL, Sigma-Aldrich, P7000, St. Louis, MO, USA) was added, and the tubes were incubated in a shaking water bath at 37 °C for 30 min. Afterward, 4 mL of sodium acetate buffer (0.5 M, pH 5.2) and 1 mL of an enzyme mixture consisting of 0.104 g pancreatin (Sigma-Aldrich; P7545, St. Louis, MO, USA) and 14.45 U amyloglucosidase (3300 U/mL; Megazyme Int. Ltd., Wicklow, Ireland) were introduced into each tube. The tubes were then incubated horizontally in a shaking water bath at 37 °C.

At 0 and 90 min, aliquots of 100 μL were collected and transferred into Eppendorf tubes, followed by the addition of 1 mL absolute ethanol. The mixtures were centrifuged at 6500 rpm for 10 min, and the glucose content of the supernatants was quantified using the glucose oxidase–peroxidase (GOPOD) reagent (Megazyme Int.) with a uv-vis spectrophotometer (Shimadzu UV-1800, Kyoto, Japan) set to 510 nm.

The hydrolysis index (*HI*), which reflects the rate of starch digestion, was calculated as the ratio of the area under the hydrolysis curve of the sample to that of the reference white bread (*GI* = 100). The *HI* was calculated using Equation (3) as follows:

(3) $$HI=\frac{Area\;under\;the\;curve\;of\;the\;sample}{Area\;under\;the\;curve\;of\;white\;bread}$$

The in vitro glycemic index (*GI*) was subsequently estimated using Equation (4) proposed by Goñi et al. [23].

(4) GI=39.71+0.549HI

### 2.10. Volatile Compound Profile

Volatile components of bread samples were detected by gas chromatography and mass spectroscopy (GC-MS) equipped with a Restec (Bellfonte, Bellefonte, PA, USA) Rtx-5MS fused silica capillary column (30 m × 0.25 mm × 0.25 µm). The solid phase micro-extraction (SPME) method was used, and bread samples of 2 g were weighed into a 20 mL headspace vial and incubated for 1 h at 60 °C. The analysis program in a previous study by Sahin et al. [24] was used: the column was held at 35 °C for 5 min, then increased to 50 °C at 5 °C/min and held for 5 min. Next, the temperature was raised to 230 °C at 5.5 °C/min and held for 5 min. The total run time was 50.73 min. Helium was used as the carrier gas at a flow rate of 1 mL/min. Mass spectra were recorded in the range of 35–650 *m*/*z* with an ionization energy of 70 eV. Volatile compounds were tentatively identified by comparison with commercial mass spectral libraries (NIST27 and WILEY7), using a minimum similarity index threshold of 50%.

### 2.11. Statistical Analysis

Analysis of variance (ANOVA) followed by Duncan’s multiple range test was conducted to determine statistically significant differences among mean values (*p* < 0.05). Statistical analyses were performed using SPSS 18.0 software (SPSS Inc., Chicago, IL, USA). Principal component analysis (PCA) was carried out using JMP Pro (v.17, SAS Inc., Cary, NC, USA) to differentiate the volatile compounds in bread samples. PCA was based on the percentage composition (area %) of the volatile compounds identified by HS/GC-MS analysis.

## 3. Results and Discussion

### 3.1. Sourdough Characteristics

**Figure 2:**
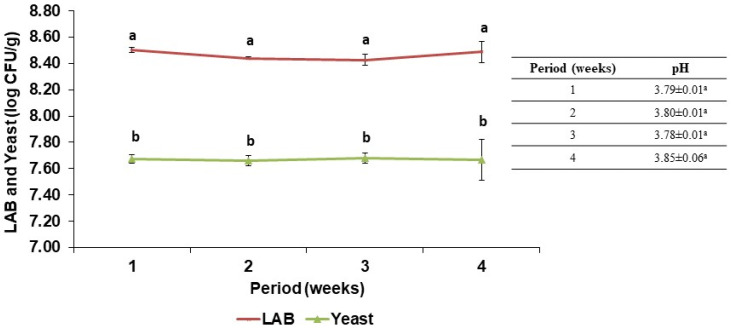
General characteristics of type I sourdough during 4 weeks of storage.

The pH and microbiological properties of the type I sourdough used in bread production were monitored during 4 weeks to ensure the stability (Figure 2). The pH values of the sourdough remained remarkably constant, ranging from 3.78 ± 0.01 to 3.85 ± 0.06, which is within the characteristic pH range reported for mature type I sourdough [25]. In the study by Vrancken et al. [26], the final pH of the sourdoughs stabilized after approximately the third back-slopping cycle and ranged from 3.3 to 3.4. A fermentation temperature of 23 °C increased the final pH to 3.7, which was similar to the value observed in the present study. Microbiological analysis revealed a balanced microbial ecosystem since LAB counts were maintained at higher levels than the yeast population (Figure 1) and remained stable throughout the four weeks. Vrancken et al. [26] reported final LAB cell densities between log 8 and log 9 CFU g^−1^ and yeast counts of approximately log 8 CFU g^−1^, with both populations varying according to fermentation temperature. Statistical analysis (*p* < 0.05) showed no significant differences in pH, LAB, or yeast counts during the storage period. This stability indicates that the sourdough was well standardized, ensuring the reproducibility of the fermentation process in the preparation of VKSB and APSB.

### 3.2. Effects of Partial Baking and Storage on Specific Volume, Baking Loss, and Textural Properties of Breads

Partial baking affected the specific volume (SV) of breads depending on bread type and storage duration (Table 1). Non-par-baked RB (day 0) exhibited the highest specific volume, while non-par-baked APSB (day 0) showed the lowest value (*p* < 0.05). With increasing par-baked frozen storage time, the reduction in specific volume was observed, particularly in RB, where values decreased significantly after 15 and 60 days. This reduction may be attributed to structural weakening of the gluten–starch matrix during frozen storage, which limits gas retention during final baking [27]. Recent studies have demonstrated that freezing induces mechanical disruption in dough structure and alters starch–protein interactions, ultimately reducing loaf volume [28]. Also, RB samples showed no significant differences between 15 and 30 days of storage (*p* = 0.856), whereas VKSB samples exhibited a significant reduction in SVs only after 60 days (*p* < 0.05). Partial baking and storage up to 60 days did not significantly affect the SVs of APSB samples (*p* = 0.142). The SV results showed that the effect of partial baking was more pronounced on RB since traditional breads exhibited no significant reduction up to 30 days for VKSB and 60 days for APSB.

Baking loss is closely associated with the ability of the dough and par-baked bread matrix to retain water during storage and rebaking. Frozen storage may alter water distribution and moisture-binding properties within the bread structure, which can affect moisture retention and consequently influence baking loss during final baking [29,30]. Baking loss decreased with increasing frozen storage time of par-baked samples up to 30 days (*p* < 0.05), followed by an increase at 60 days, which was particularly pronounced in the VKSB sample (*p* < 0.05). Previous studies have reported that baking loss in rebaked bread can be influenced by initial baking conditions and storage duration prior to final baking [31]. Therefore, the increase in baking loss observed in VKSB at day 60 may be associated with storage-induced changes affecting moisture retention during rebaking. This result could also be explained by the higher moisture content of VKSB at day 60 compared with the other samples (see Table 3 in Section 3.3). Higher moisture levels may lead to greater water loss during thawing and baking, resulting in increased baking loss [30]. Karaoğlu and Kotancılar [31] also reported that storage duration prior to final baking can lead to variations in baking loss.

Hardness values of par-baked breads increased significantly after 60 days of frozen storage for all bread types (Table 2). RBs and VKSBs exhibited the highest hardness values, whereas APSB showed a comparatively softer crumb structure after 60 days of storage. Chewiness followed a trend similar to hardness, increasing significantly after prolonged storage prior to final baking. Cohesiveness and resilience decreased gradually with frozen storage time prior to final baking, indicating loss of crumb elasticity [32]. APSB exhibited a lower hardness and higher resilience value when compared to RB. Zhai et al. [33] reported a reduction in bread with mashed potatoes at 30% addition level, and this reduction was attributed to weakening the gluten–starch interactions.

### 3.3. Effects of Partial Baking and Storage on the Moisture Content and the Water Evaporation Enthalpy of Breads

The effects of partial baking and storage prior to final baking on the moisture content of breads are presented in Table 3. Significant reduction was observed during frozen storage prior to final baking, except for VKSB. Also, VKSB exhibited higher moisture content when compared to other breads, and no significant effect of frozen storage prior to final baking on its moisture content was shown. In a study of Kotancilar et al. [34], traditional VKB exhibited a higher crust and crumb moisture than white bread. This property of VKB was attributed to its hard and thick crust characteristics. The moisture content of APSB remained partially stable, showing no significant reduction after 15 days of storage (*p* = 0.162). This can be due to the usage of mashed potatoes in the formulation. Mashed potatoes can hold high moisture content, unlike the other potato-derived components [33].

The changes in the thermal behavior of RB, APSB, and VKSB samples during par-baked frozen storage (0, 15, 30, and 60 days) are also presented in Table 3. Differential scanning calorimetry (DSC) data provide critical parameters for understanding the effects of starch gelatinization, amylopectin crystallization, and retrogradation processes on storage stability [35]. Par-baking and par-baked storage at −18 °C prior to final baking caused a reduction in the enthalpy associated with water evaporation (day 0, *p* < 0.05). The reduction was dependent on par-baked storage time and bread type. This trend in enthalpy values can be attributed to the specific impact of par-baking and par-baked storage on the starch structure [36]. Non-par-baked VKSB sample (day 0) showed the highest enthalpy value when compared to other non-par-baked samples (day 0). This may be due to the longer fermentation time used in VKSB production. Longer fermentation time led to an increase in sugar levels as the starch was degraded [15,37]. Sugar arising may have altered the binding properties of water, and this may have affected the enthalpy value [21]. The lowest enthalpy value was obtained for APSB subjected to 30 and 60 days of par-baked storage (Table 3). This finding may be associated with structural modifications caused by the potato incorporated into the formulation, as well as by par-baked frozen storage. These factors may have led to a different starch–water reorganization compared to wheat-only formulations [36].

### 3.4. Staling Properties of Breads After Partial Baking Storage

**Figure 3:**
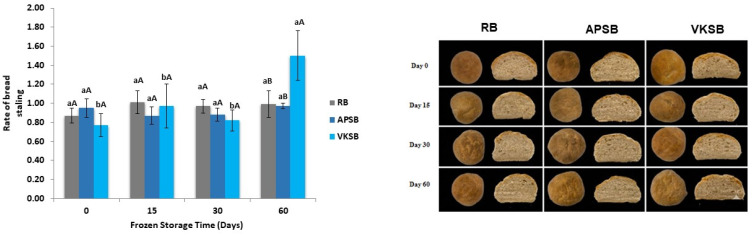
Effect of partial baking and frozen storage on the staling rate of breads. Different lowercase letters indicate statistically significant differences during the par-baked frozen storage period of the same bread type (*p* < 0.05). Different uppercase letters indicate statistically significant differences between bread types at the same par-baked frozen storage time (*p* < 0.05). RB: regular bread; APSB: Afyonkarahisar potato style bread; VKSB: Vakfıkebir style bread.

Crumb hardness is widely regarded as one of the most reliable indicators of bread staling [3]. From this point of view, the staling rate was calculated based on the hardness values of the bread samples after final baking during 5 days of storage at room temperature, as shown in Table 4 and presented in Figure 3. The hardness values of all bread samples increased significantly during the storage period (*p* < 0.05). As for the staling rate, no significant differences were observed among the non-par-baked samples (day 0) during 5 days of storage at room temperature (*p* = 0.078). Similarly, breads that were par-baked and stored at −18 °C prior to final baking showed no significant differences in staling rate, except for VKSB (Figure 2) (*p* = 0.165 and *p* = 0.185). A 60-day par-baked frozen storage period increased the staling rate of VKSB compared to the 15- and 30-day par-baked frozen storage periods. This finding indicates that a prolonged storage period influenced the staling rate of VKSB more than the partial baking process itself. APSB exhibited a lower staling rate after a 60-day par-baked storage period than VKSB, and its staling rate remained stable throughout the par-baked frozen storage periods (Figure 2). This may be attributed to the crystallinity of starch [38].

Storage of breads after final baking caused pronounced changes in other textural parameters across all bread types (Table 4). Cohesiveness is a useful indicator that provides information about bread staling properties. It indicates the strength of the internal bonds holding the crumb together and reflects the bread’s resistance to crumbling. High cohesiveness values are desirable in bread quality, as they indicate reduced crumbling during storage [32]. A reduction in cohesiveness was observed during 5 days of storage, regardless of bread type. A sharp reduction was shown on day 3 of storage, especially for non-par-baked RB (Table 4). In contrast, this reduction occurred on day 1 in par-baked RB stored for 15 days prior to final baking, and par-baked APSB and VKSB exhibited higher cohesiveness value than RB. Sourdough fermentation used in APSB and VKSB may lead to lower crumbliness [39]. Resilience, another critical indicator of bread freshness, reflects the ability of the crumb to recover from a given deformation [40]. A significant reduction in this parameter was also observed during storage. Higher resilience values were obtained in non-par-baked APSB, indicating its ability to retain mechanical strength. However, the partial baking process caused a pronounced reduction in resilience for all bread types.

### 3.5. Estimated Glycemic Index (eGI) Changes

**Figure 4:**
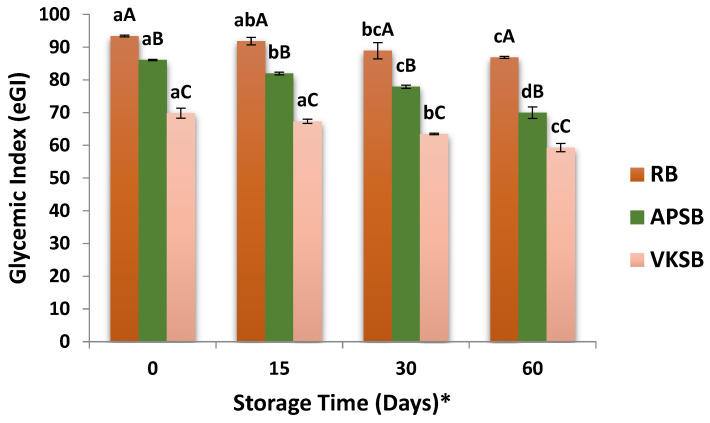
Effect of partial baking storage on the estimated glycemic index of breads. Different lowercase letters indicate statistically significant differences during the par-baked frozen storage period of the same bread type (*p* < 0.05). Different uppercase letters indicate statistically significant differences between bread types at the same par-baked frozen storage time (*p* < 0.05). RB: regular bread; APSB: Afyonkarahisar potato style bread; VKSB: Vakfıkebir style bread. * Storage time day 0 means non-par-baked samples.

The effect of par-baking and frozen storage at −18 °C prior to final baking on the eGI of breads was shown in Figure 4. Among the samples, VKSB consistently exhibited the lowest eGI values for both the non-par-baked group (storage time 0) and the par-baked group. It was lower than 70, which corresponds to foods classified as having a medium GI [41]. Both traditional breads showed lower eGI values compared to RB, which can be attributed to the sourdough effect [42]. Par-baking led to a gradual reduction in the eGI of APSB throughout all storage periods prior to final baking, whereas VKSB and RB showed this reduction only after 30 and 60 days of storage. APSB showed the greatest reduction in eGI during storage, decreasing from 86.1 at day 0 to 69.9 after 60 days of storage. This reduction in eGI is mainly attributed to the increased formation of resistant starch during storage, as starch molecules reorganize into more ordered structures that are less digestible [3,7,43]. Similar processing approaches, including frozen storage prior to final baking, have also been proposed as strategies to reduce the glycemic index of wheat bread products [44]. Consistent with this, storage-induced starch retrogradation in wheat bread systems has been associated with increased resistant starch formation and reduced starch digestibility due to limited enzymatic accessibility [35]. The presence of potato in bread formulation also decreased the eGI value, but still it was classified as a high glycemic index food (GI > 75) in a study of Whitney and Simsek [45], similar to our APSB sample.

### 3.6. Volatile Compound Profiles of Breads

**Figure 5:**
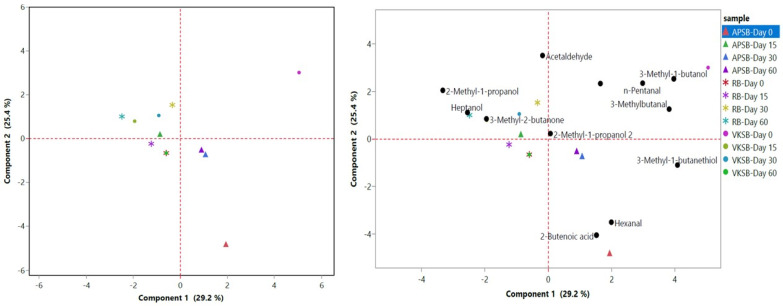
PCA plot volatile compound profiles of bread samples. RB: regular bread; APSB: Afyonkarahisar potato style bread; VKSB: Vakfıkebir style bread.

Principal component analysis (PCA) was conducted to evaluate the effect of par-baking on the volatile compound profiles of bread samples during storage (Figure 5). The first two principal components (PC1 and PC2) together explained 54.6% of the total variance, with PC1 accounting for 29.2% and PC2 accounting for 25.4% of the variability, respectively.

Only volatile compounds that have been consistently reported in the literature were selected for PCA, which may have contributed to the relatively low proportion of explained variance. Nevertheless, non-par-baked breads (VKSB-Day 0 and APSB-Day 0) were clearly separated from regular and par-baked breads, suggesting that these traditional breads possess distinctive volatile compound profiles (Figure 5). The VKSB-Day 0 sample was clearly separated from the other samples along the positive side of PC1 and PC2, suggesting a distinctive set of volatile compounds characterized by 2-methylbutanal and 3-methylbutanal. APSB-Day 0 was located on the negative side of PC2, indicating a distinct volatile profile, with hexanal and 1-pentanol being the primary contributors to this separation. The remaining samples were mainly clustered around the center of the plot, reflecting relatively similar volatile characteristics. The close positioning of those samples indicated that frozen storage after par-baking caused moderate changes in the volatile compound profile. However, the separation of fresh samples, particularly VKSB-Day 0 and APSB-Day 0, suggested that bread type had a stronger influence on the volatile profile. Overall, PCA results demonstrated that both bread type and frozen storage after par-baking influenced the development of volatile compounds, while the volatile compound profile of each par-baked traditional bread type was generally preserved throughout storage.

## 4. Conclusions

Par-baking technology is already widely applied in industrial bread production. In this study, the effects of partial baking method and frozen storage prior to final baking on bread types similar to traditional Turkish breads (Afyonkarahisar potato bread and Vakfıkebir bread) were evaluated in terms of bread quality, estimated glycemic index (eGI), and volatile compound profile in order to increase their availability, consumption, and product diversity. Afyonkarahisar potato style bread (APSB) and Vakfıkebir style bread (VKSB) possess distinct volatile compound profiles. Principal component analysis indicated clear differentiation in volatile compound profiles between non-par-baked breads (VKSB-Day 0 and APSB-Day 0) and both regular and par-baked breads. APSB and VKSB exhibited no significant reduction in specific volume during 30 days of storage, indicating that they may be more suitable for partial baking technology than regular bread with respect to specific volume. Crumb hardness of APSB and VKSB generally remained stable during 30 days of storage, whereas some variations were observed in regular bread. Crumb hardness was monitored for 5 days after final baking, and all bread samples showed no significant differences in staling rate after up to 30 days of frozen par-baked storage prior to final baking. These findings suggest that frozen par-baked storage can be applied to all bread types in this study for up to 30 days. However, APSB and VKSB were more favorable, as they provided lower eGI in addition to exhibiting a significant reduction during prolonged frozen par-baked storage. Further studies are needed to optimize par-baking, storage, and thawing conditions in order to minimize quality losses and improve product quality.

## Figures and Tables

**Table 1:** Effects of partial baking on the specific volume and baking loss of breads.

		Bread Type		
	Par-Baked Storage Time (Day) *	RB	APSB	VKSB
Specific Volume (mL/g)	0	4.43 ± 0.02 ^aA^	3.81 ± 0.23 ^aB^	4.17 ± 0.26 ^aAB^
	15	3.75 ± 0.26 ^bA^	3.78 ± 021 ^aA^	3.95 ± 0.03 ^aA^
	30	3.73 ± 0.21 ^bA^	3.63 ± 0.20 ^aA^	3.91 ± 0.06 ^aA^
	60	3.38 ± 0.13 ^cA^	3.55 ± 0.06 ^aA^	3.43 ± 0.13 ^bA^
Baking loss (%)	0	17.88 ± 0.31 ^aA^	16.45 ± 1.41 ^aA^	16.41 ± 1.15 ^bA^
	15	15.96 ± 1.78 ^abA^	15.88 ± 0.58 ^aA^	15.37 ± 0.19 ^bA^
	30	13.25 ± 0.82 ^cA^	13.42 ± 0.67 ^bA^	12.64 ± 1.25 ^cA^
	60	14.29 ± 065 ^bcB^	14.87 ± 0.51 ^abB^	18.34 ± 0.91 ^aA^

Different lowercase letters indicate statistically significant differences during the par-baked frozen storage period of the same bread type (*p* < 0.05). Different uppercase letters indicate statistically significant differences between bread types at the same par-baked frozen storage time (*p* < 0.05). RB: regular bread; APSB: Afyonkarahisar potato style bread; VKSB: Vakfıkebir style bread. * Par-baked storage time day 0 means non-par-baked samples.

**Table 2:** Effects of partial baking storage on the textural properties of breads.

		Bread Type		
	Par-Baked Storage Time (Day) *	RB	APSB	VKSB
Hardness (N)	0	1.43 ± 0.13 ^bA^	1.03 ± 0.02 ^bB^	1.06 ± 0.04 ^bB^
	15	1.54 ± 0.35 ^bA^	1.03 ± 0.03 ^bB^	1.05 ± 0.03 ^bB^
	30	1.18 ± 0.22 ^bA^	1.02 ± 0.01 ^bA^	1.08 ± 0.07 ^bA^
	60	2.48 ± 0.29 ^aA^	1.60 ± 0.13 ^aB^	2.24 ± 0.33 ^aA^
Cohesiveness	0	0.90 ± 0.01 ^aA^	0.91 ± 0.01 ^aA^	0.91 ± 0.01 ^aA^
	15	0.86 ± 0.01 ^bAB^	0.88 ± 0.02 ^bA^	0.85 ± 0.01 ^bB^
	30	0.84 ± 0.01 ^cA^	0.85 ± 0.01 ^cA^	0.86 ± 0.01 ^bcA^
	60	0.82 ± 0.01 ^cB^	0.84 ± 0.01 ^cB^	0.87 ± 0.01 ^cA^
Chewiness (N)	0	1.06 ± 0.01 ^bB^	1.38 ± 0.01 ^bA^	1.08 ± 0.10 ^bB^
	15	1.07 ± 0.19 ^bA^	1.42 ± 0.44 ^bA^	1.24 ± 0.23 ^bA^
	30	1.36 ± 0.59 ^bA^	1.40 ± 0.22 ^bA^	1.36 ± 0.36 ^bA^
	60	2.84 ± 1.08 ^aA^	2.42 ± 0.10 ^aA^	2.67 ± 0.38 ^aA^
Resilience	0	0.62 ± 0.01 ^aB^	0.65 ± 0.01 ^aA^	0.63 ± 0.01 ^aB^
	15	0.54 ± 0.03 ^bA^	0.50 ± 0.01 ^bB^	0.55 ± 0.01 ^bA^
	30	0.45 ± 0.01 ^dB^	0.46 ± 0.01 ^cB^	0.48 ± 0.01 ^cA^
	60	0.48 ± 0.01 ^cC^	0.51 ± 0.01 ^bB^	0.57 ± 0.02 ^bA^

Different lowercase letters indicate statistically significant differences during the par-baked frozen storage period of the same bread type (*p* < 0.05). Different uppercase letters indicate statistically significant differences between bread types at the same par-baked frozen storage time (*p* < 0.05). RB: regular bread; APSB: Afyonkarahisar potato style bread; VKSB: Vakfıkebir style bread. * Par-baked storage time day 0 means non-par-baked samples.

**Table 3:** Effects of partial baking on the moisture content and differential scanning calorimetry (DSC) of breads.

		Bread Type		
	Par-Baked Storage Time (Day) *	RB	APSB	VKSB
Moisture content (%)	0	40.50 ± 0.01 ^aAB^	40.20 ± 0.13 ^aB^	40.61 ± 0.16 ^aA^
	15	38.67 ± 0.01 ^bA^	38.92 ± 0.01 ^bA^	39.87 ± 0.04 ^aA^
	30	38.61 ± 0.01 ^bB^	38.80 ± 0.01 ^bcB^	40.33 ± 0.44 ^aA^
	60	38.53 ± 0.04 ^cB^	38.71 ± 0.01 ^cB^	40.38 ± 0.54 ^aA^
Enthalpy (J/g)	0	456.00 ± 1.41 ^aB^	454.50 ± 1.71 ^aB^	479.50 ± 1.36 ^aA^
	15	442.65 ± 1.32 ^bA^	442.25 ± 1.96 ^aA^	425.40 ± 1.71 ^bA^
	30	440.05 ± 1.07 ^bA^	405.00 ± 1.07 ^bC^	424.95 ± 1.34 ^bB^
	60	432.50 ± 1.71 ^cA^	401.55 ± 1.78 ^bC^	423.35 ± 1.91 ^bB^

Different lowercase letters indicate statistically significant differences during the par-baked frozen storage period of the same bread type (*p* < 0.05). Different uppercase letters indicate statistically significant differences between bread types at the same par-baked frozen storage time (*p* < 0.05). RB: regular bread; APSB: Afyonkarahisar potato style bread; VKSB: Vakfıkebir style bread. * Par-baked storage time day 0 means non-par-baked samples.

**Table 4:** Changes in the textural properties of fully baked breads during storage.

			Bread Type		
	Par-Baked Storage Time (Day) *	Storage Time of Final Bread (Day)	RB	APSB	VKSB
Hardness (N)	0	0	1.43 ± 0.13 ^t^	1.03 ± 0.02 ^t^	1.06 ± 0.04 ^z^
		1	2.49 ± 0.29 ^z^	2.50 ± 0.83 ^z^	1.26 ± 0.17 ^z^
		3	4.65 ± 0.73 ^y^	3.64 ± 0.18 ^y^	3.44 ± 0.35 ^y^
		5	5.79 ± 0.38 ^x^	5.76 ± 0.53 ^x^	4.89 ± 0.59 ^x^
	15	0	1.54 ± 0.35 ^z^	1.03 ± 0.06 ^t^	1.05 ± 0.03 ^z^
		1	2.48 ± 0.31 ^y^	2.03 ± 0.40 ^z^	1.53 ± 0.18 ^z^
		3	2.54 ± 0.19 ^y^	3.29 ± 0.86 ^y^	3.17 ± 0.07 ^y^
		5	6.61 ± 0.37 ^x^	5.39 ± 0.27 ^x^	5.91 ± 0.16 ^x^
	30	0	1.18 ± 0.22 ^t^	1.02 ± 0.01 ^t^	1.08 ± 0.07 ^z^
		1	2.21 ± 0.57 ^z^	2.19 ± 0.30 ^z^	1.79 ± 0.65 ^z^
		3	4.44 ± 0.41 ^y^	3.56 ± 0.22 ^y^	4.24 ± 0.32 ^y^
		5	6.03 ± 0.16 ^x^	5.43 ± 0.36 ^x^	5.18 ± 0.54 ^x^
	60	0	2.48 ± 0.29 ^z^	1.60 ± 0.13 ^t^	2.24 ± 0.33 ^z^
		1	2.63 ± 0.33 ^z^	2.59 ± 0.05 ^z^	2.67 ± 0.81 ^z^
		3	5.10 ± 0.10 ^y^	4.07 ± 0.11 ^y^	6.05 ± 0.05 ^y^
		5	7.43 ± 0.41 ^x^	6.45 ± 0.26 ^x^	9.74 ± 0.21 ^x^
Cohesiveness	0	0	0.90 ± 0.01 ^x^	0.91 ± 0.01 ^x^	0.91 ± 0.01 ^x^
		1	0.87 ± 0.01 ^y^	0.88 ± 0.01 ^y^	0.88 ± 0.01 ^x^
		3	0.77 ± 0.01 ^z^	0.83 ± 0.01 ^z^	0.79 ± 0.02 ^y^
		5	0.75 ± 0.01 ^t^	0.73 ± 0.01 ^t^	0.74 ± 0.04 ^z^
	15	0	0.86 ± 0.01 ^x^	0.88 ± 0.86 ^x^	0.85 ± 0.01 ^x^
		1	0.76 ± 0.01 ^y^	0.82 ± 0.84 ^y^	0.81 ± 0.01 ^y^
		3	0.74 ± 0.01 ^y^	0.73 ± 0.74 ^z^	0.73 ± 0.01 ^z^
		5	0.70 ± 0.08 ^y^	0.72 ± 0.073 ^z^	0.70 ± 0.02 ^z^
	30	0	0.84 ± 0.01 ^x^	0.85 ± 0.01 ^x^	0.86 ± 0.01 ^x^
		1	0.78 ± 0.01 ^y^	0.73 ± 0.01 ^y^	0.83 ± 0.02 ^x^
		3	0.71 ± 0.03 ^z^	0.68 ± 0.03 ^z^	0.73 ± 0.02 ^y^
		5	0.68 ± 0.03 ^z^	0.65 ± 0.01 ^z^	0.69 ± 0.02 ^z^
	60	0	0.82 ± 0.01 ^x^	0.84 ± 0.01 ^x^	0.87 ± 0.01 ^x^
		1	0.77 ± 0.01 ^y^	0.78 ± 0.03 ^x^	0.81 ± 0.01 ^y^
		3	0.70 ± 0.01 ^z^	0.60 ± 0.09 ^y^	0.72 ± 0.01 ^z^
		5	0.59 ± 0.03 ^t^	0.59 ± 0.01 ^y^	0.65 ± 0.02 ^t^
Resilience	0	0	0.62 ± 0.01 ^x^	0.65 ± 0.01 ^x^	0.63 ± 0.01 ^x^
		1	0.57 ± 0.01 ^y^	0.61 ± 0.01 ^y^	0.58 ± 0.01 ^y^
		3	0.44 ± 0.01 ^z^	0.56 ± 0.01 ^z^	0.45 ± 0.01 ^z^
		5	0.42 ± 0.01 ^t^	0.47 ± 0.01 ^t^	0.43 ± 0.02 ^z^
	15	0	0.54 ± 0.03 ^x^	0.50 ± 0.49 ^x^	0.55 ± 0.01 ^x^
		1	0.40 ± 0.01 ^y^	0.46 ± 0.48 ^y^	0.49 ± 0.01 ^y^
		3	0.35 ± 0.01 ^yz^	0.37 ± 0.39 ^z^	0.39 ± 0.01 ^z^
		5	0.33 ± 0.06 ^z^	0.35 ± 0.36 ^z^	0.38 ± 0.02 ^z^
	30	0	0.45 ± 0.01 ^x^	0.46 ± 0.01 ^x^	0.48 ± 0.01 ^x^
		1	0.36 ± 0.01 ^y^	0.33 ± 0.01 ^y^	0.45 ± 0.02 ^y^
		3	0.30 ± 0.01 ^z^	0.30 ± 0.01 ^z^	0.38 ± 0.02 ^z^
		5	0.29 ± 0.01 ^z^	0.29 ± 0.01 ^z^	0.35 ± 0.01 ^t^
	60	0	0.48 ± 0.01 ^x^	0.51 ± 0.01 ^x^	0.57 ± 0.02 ^x^
		1	0.40 ± 0.01 ^y^	0.43 ± 0.02 ^y^	0.49 ± 0.01 ^y^
		3	0.28 ± 0.02 ^z^	0.31 ± 0.01 ^z^	0.36 ± 0.01 ^z^
		5	0.26 ± 0.02 ^z^	0.27 ± 0.01 ^t^	0.33 ± 0.02 ^z^

Different lowercase letters indicate statistically significant differences within the same bread type and the same par-baked storage time during the fully baked storage period (*p* < 0.05). RB: regular bread; APSB: Afyonkarahisar potato style bread; VKSB: Vakfıkebir style bread. * Par-baked storage time day 0 means non-par-baked samples.

## Data Availability

The original contributions presented in the study are included in the article; further inquiries can be directed to the corresponding author.
